# Early Detection of Tibial Cartilage Degradation and Cancellous Bone Loss in an Ovariectomized Rat Model

**DOI:** 10.1155/2017/9654056

**Published:** 2017-01-15

**Authors:** Yinong Wang, Zhiwei Liu, Qing Wang, Qianjin Feng, Wufan Chen

**Affiliations:** ^1^Institute of Medical Information, School of Biomedical Engineering, Southern Medical University, Guangzhou 510515, China; ^2^Guangdong Provincial Key Laboratory of Medical Image Processing, Southern Medical University, Guangzhou 510515, China

## Abstract

This study aimed to investigate degradation of the articular cartilage and loss of the cancellous bone in an ovariectomized (OVX) rat model simulating early human menopausal stage. Fourteen health female Sprague-Dawley rats were randomly divided into two groups (*n* = 7 per group): an OVX group that underwent bilateral ovariectomy to create an OVX model with low estrogen levels and a sham group in which only the periovarian fatty tissue was exteriorized. All the animals were sacrificed at 3 weeks after ovariectomy. The left tibiae were harvested. The articular cartilage at medial tibial plateau (MTP) and lateral tibial plateau (LTP) was assessed with quantitative high-frequency ultrasound. The cancellous bone was evaluated with micro-CT. The results indicated that, in comparison with the sham rats, the OVX rats exhibited significant alterations in acoustic parameters of the articular cartilage but insignificant changes in microarchitectural parameters of the cancellous bone in early stage of low estrogen levels. The results of this study suggest that cartilage degradation induced by estrogen reduction was detected earlier with quantitative ultrasound than that of the cancellous bone loss in 3 wk OVX rats.

## 1. Introduction

Osteoporosis (OP) and osteoarthritis (OA) are severe progressive diseases with high prevalence in the elderly and affect both men and women. However, the incidence of OP and OA increases in menopausal women. Main consequence of OP is the increased risk of bone fractures due to abnormalities in amount and microarchitectural arrangement of bone tissue. OA, on the other hand, causes pain and disability of one or many synovial joints resulted from progressive degradation of the articular cartilage with alterations in the bone and surrounding structures. These two diseases play a negative role in the longevity and health of menopausal women.

Both the bone and articular cartilage are the target tissues of estrogens. It has been found that estrogen receptors (ER) such as ER*α* and ER*β* exist in osteoclasts, osteoblasts, and articular chondrocytes [1–3]. Estrogen deficiency exacerbates bone resorption exceeding bone formation resulting in menopausal OP [4–6]. The low estrogen level induces a faster loss of knee joint cartilage in menopausal women than in men of the same age [7]. The degradation of articular cartilage is one of indications of the early OA and can trigger the occurrence of menopausal OA [8].

Therefore, the menopause-related changes in estrogen levels affect generation of both bone and articular cartilage and thus induce the pathogenesis of OP and OA. Due to their high prevalence in elderly women, it could be anticipated that OP and OA coexist frequently. However, a defined relationship between OP and OA has not yet clearly delineated. There are conflicting findings. Some studies reported that OA was associated with higher bone mineral density (BMD), that is, an inverse relationship between OA and OP [9–11]. Some studies suggested a positive association between OP and OA that bone loss occurred with cartilage loss in OA patients [12, 13]. One study interestingly found that both high and low BMD conditions could induce OA [14]. Others did not find a relation between cartilage degradation and bone loss [15].

Furthermore, the occurrence of OA and OP is found to be of site-relevance [9]. However, the clearly defined site-relationship between OA and OP is still debatable. Okano et al. found that end-stage hip OA induced lower BMD in the calcaneus but higher BMD in the spine and radius [16]. Lethbridge-Çejku et al. reported that knee OA patients had higher BMD in spine but not in hip [17], while Multanen et al. suggested that knee OA menopausal women had higher hip bone strength [11]. Therefore, more investigations are needed to delineate associations between cartilage degradation and bone loss at different anatomic locations.

To effectively evaluate BMD, dual-energy X-ray absorptiometry (DXA) has been widely used [10, 11, 16]. DXA, however, provides little information of bone structure. Microcomputed tomography (micro-CT), a high resolution imaging tool, is used in quantitative assessments of osteoporotic alterations and therapeutic improvements in microarchitectural characteristics of the trabecular and cortical bone in the OP animal model [18–20]. Quantitative ultrasound, on the other hand, is used to characterize the degeneration of articular cartilage [21–23].

Therefore, the present study focused on menopausal alterations in the bone and articular cartilage in the tibia. We applied bilateral ovariectomy to create an OVX rat model with low estrogen levels and then assessed early-stage alterations in the bone and articular cartilage with micro-CT and quantitative ultrasound.

## 2. Materials and Methods

### 2.1. Animal Care and Experimental Protocol

Fourteen female Sprague-Dawley rats, aged 10 months old and weighting 299.1 to 383.8 g, were used in this study. They were purchased from Guangdong Medical Laboratory Animal Center, China, and individually kept in metal cages under 12-hour light-dark cycle with standard rat diet and water ad libitum. The animals were randomly divided into two groups (*n* = 7 per group): an OVX group that underwent bilateral ovariectomy to create an OVX model with low estrogen levels and a sham group in which only the periovarian fatty tissue was exteriorized. After ovariectomy, the animals were returned to their cages and fed according to the aforementioned feeding protocol. Before conducting the experiments, ethical approval of this study (SYXK[Yue]2008-0002) was obtained from the Animal Experimental Ethical Inspection Committee of Guangdong Medical Laboratory Animal Center, China. Experiments on rats were performed in accordance with the Guidelines for the Care of Laboratory Animals of the National Institutes of Health.

All animals, weighting 309.9 to 423.3 g, were euthanized with an overdose of sodium pentobarbital (P3761, Sigma, USA) 3 weeks after ovariectomy. The left tibias were excised, harvested, and stored at −20°C until the ultrasound examination.

### 2.2. Ultrasound Examinations

**Figure 1:**
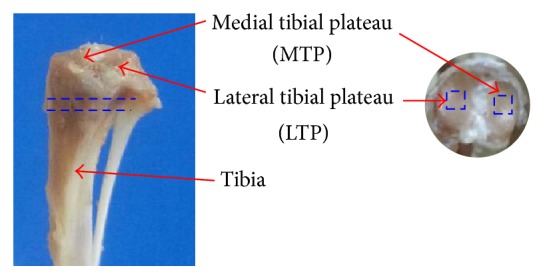
Schematic of the areas scanned by ultrasound biomicroscopy* (two blue dotted squares 0.3 mm × 0.3 mm on the medial and lateral tibial plateaus)* and micro-CT* (two blue dotted lines presenting the volume of interest of the tibial metaphysis, approximately 0.1 mm in thickness)*.

Before ultrasound scanning, the tibial sample to be measured was thawed in a saline solution for 2 hours. Then, the specimen was vertically fixed with a clamp and immersed in saline in the container. A square region of 0.3 mm × 0.3 mm on the top surface of cartilage tissue from the medial tibial plateau (MTP) and lateral tibial plateau (LTP) was, respectively, selected as region of interest (ROI) (Figure 1) and perpendicularly placed to the ultrasound beam.

The ultrasound system was comprised of an ultrasound pulser/receiver (Olympus 5900 PR, Panametrics-NDT, Waltham, MA, USA), a 50 MHz transducer (Olympus, PI50-2-R0.75, Panametrics-NDT), and a computer with a 12-bit A/D acquisition card (CompuScope 12400, Gage, ON, Canada). The ultrasound scanning over ROI was controlled by custom-developed software to move the transducer with a 3D translating stage (ETSN400, Tian-Rui-Zhong-Hai Instrument, Beijing, China). After the transducer was properly positioned approximately 1 cm above the cartilage tissue, the ultrasound examination started. The ultrasound radiofrequency (RF) signals were saved for offline parameter extraction.

Four parameters were then automatically extracted from ultrasound RF signals processed using a self-developed MATLAB program (The MathWorks, Natick, MA, USA): ultrasound roughness index (URI) of the cartilage surface, reflection coefficient of the cartilage surface (RC_1_), reflection coefficient of the cartilage-subchondral bone interface (RC_2_), and the thickness (*h*) of the cartilage tissue. These parameters were defined by (1)–(4), respectively.

(1) URI=1m∑i=1mdi−d¯2

(2) RC1=1m∑i=1mAiAref×100%

(3) RC2=1m∑j=1mAjAref×100%

(4) h=1m∑i=1mCcartilage×TOFi2.

In these equations, *m* is the total number of the sampling lines that is equal to 100 in this study. In (1), *d*_*i*_ represents the distance from the transducer surface to the cartilage surface in the sampling line *i* and $\overset{-}{d}$ is the mean of *d*_*i*_. In (2) and (3), *A*_*i*_, *A*_*j*_, and *A*_ref_, respectively, represent the peak-to-peak amplitude of the echoes from the cartilage surface in the sampling line *i*, from the cartilage-bone interface in sampling line *j*, and from a perfect reflector. In (4), TOF_*i*_ is the time-of-flight from the cartilage surface to the cartilage-bone interface in the sampling line *i*, and *C*_cartilage_ is the average sound speed (equal to 1675 m/s) in the cartilage.

### 2.3. Micro-CT Measurements

After ultrasound examinations, the tibial samples were fixed in 4% paraformaldehyde solution for more than 72 hours. Then, 3D microarchitecture of the cancellous bone was assessed using a micro-CT system (*μ*CT80, Scanco Medical, AG, Switzerland) with energy settings of 55 kV and 145 *μ*A. The proximal tibial metaphysis was scanned starting at approximately 1 mm distal to the growth plate (Figure 1). A total of 100 consecutive tomographic CT slices were taken from proximal to distal with thickness of 0.01 mm per slice.

In order to analyze the cancellous part of the tibial metaphysis, the trabecular area was contoured manually slice by slice. Then, the trabecular volume of interest (VOI) of the tibial metaphysis was reconstructed by a reconstruction software (Scanco Medical, AG, Switzerland). The micro-CT analysis was performed, giving an isotropic voxel size of 12 *µ*m. Trabecular number (Tb.N), trabecular thickness (Tb.Th), trabecular separation/spacing (Tb.Sp), connectivity density (Conn.D.), and structure model index (SMI) were extracted to characterize the trabecular microarchitecture of the cancellous bone in menopausal rats. Moreover, BV/TV, which stands for mineralized bone volume over total volume of the given VOI, was used to evaluate relative changes in bone volume density.

### 2.4. Statistical Analysis

Statistical analyses were performed with SPSS Statistics (Version 20, IBM, Armonk, NY, USA). All values in the text were presented as mean ± standard deviation (SD). The statistical differences in the acoustic parameters and the micro-CT parameters between the OVX and sham groups were analyzed using Mann–Whitney* U *test. Significant differences were accepted with *p* ≤ 0.05.

## 3. Results

### 3.1. Results of Ultrasound Examinations

**Figure 2:**
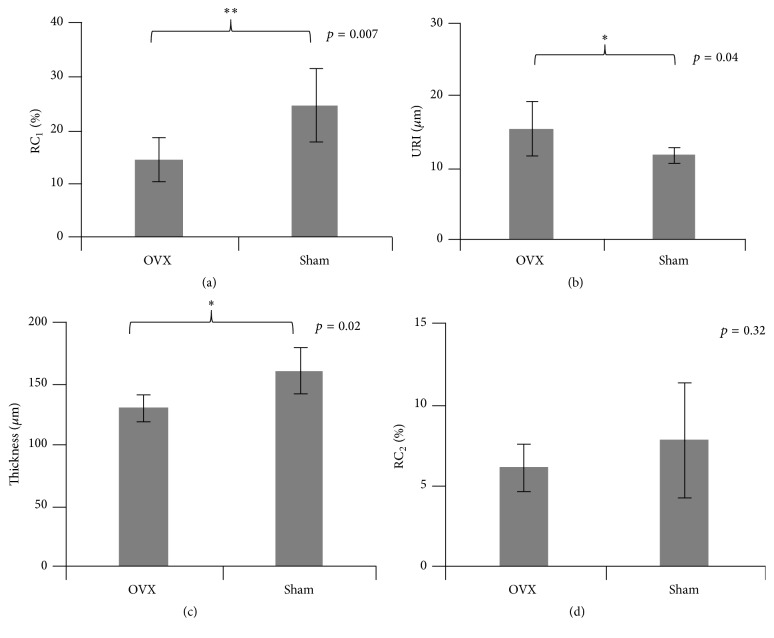
Comparisons of ultrasound results measured at medial tibial plateau (MTP) between the OVX and sham groups. (a) Reflection coefficient (RC_1_) of the cartilage surface; (b) ultrasound roughness index (URI); (c) cartilage thickness (*h*); and (d) reflection coefficient (RC_2_) of the cartilage-bone interface. ^*∗*^Statistically significant difference at level *p* < 0.05 exists between OVX group and sham group. ^*∗∗*^Statistically significant difference at level *p* < 0.01 exists between OVX group and sham group.

From the results of ultrasound examination of the articular cartilage at MTP (Figure 2), we found significant alterations in the surface cartilage tissue in the OVX group. In comparison with the sham group, RC_1_ of the OVX group significantly decreased (*p* < 0.01). URI of the OVX group significantly increased (*p* < 0.05) indicating that the surface of articular cartilage became rougher in the early-stage of low estrogen levels. Furthermore, the cartilage thickness in the OVX rats significantly decreased (*p* < 0.05). However, no significant alteration (*p* > 0.05) was found in RC_2_, which is related to the property of the cartilage-subchondral bone interface.

**Figure 3:**
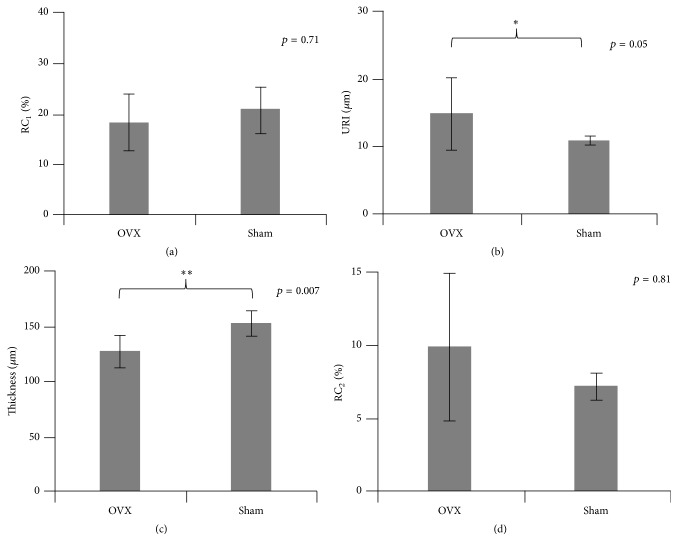
Comparisons of ultrasound results measured at lateral tibial plateau (LTP) between the OVX and sham groups. (a) Reflection coefficient (RC_1_) of the cartilage surface; (b) ultrasound roughness index (URI); (c) cartilage thickness (*h*); and (d) reflection coefficient (RC_2_) of the cartilage-bone interface. ^*∗*^Statistically significant difference at level* p* < 0.05 exists between OVX group and sham group. ^*∗∗*^Statistically significant difference at level *p* < 0.01 exists between OVX group and sham group.

Similar to the findings of MTP, significant alterations in the surface cartilage tissue at LTP were observed in the OVX group compared with the sham group (Figure 3). The significant changes in URI (*p* = 0.05) and cartilage thickness (*p* < 0.01) were observed in the OVX group. However, no significant changes (*p* > 0.05) in RC_1_ and RC_2_ were found in the OVX group compared with the sham group.

### 3.2. Results of Micro-CT Scanning

**Figure 4:**
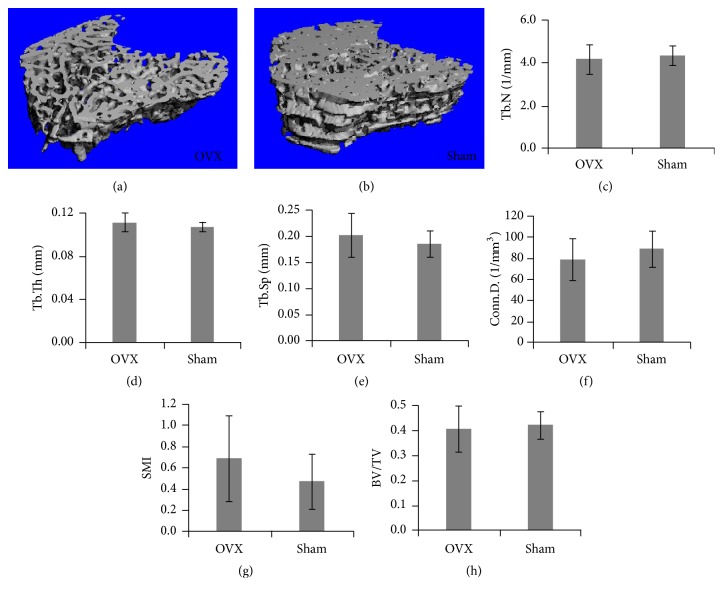
Comparisons of micro-CT results measured at the cancellous bone between the OVX and sham groups. (a) 3D reconstruction of the trabecular bone of the OVX group; (b) 3D reconstruction of the trabecular bone of the sham group: (c) Tb.N; (d) Tb.Th; (e) Tb.Sp; (f) Conn.D.; (g) SMI; (h) BV/TV.

The 3D reconstruction of the trabecular bone structures in the proximal tibial metaphysis by micro-CT is shown in Figures 4(a) and 4(b). The remarkable change of cancellous bone was not observed between the sham and OVX rats, although some of the connecting rods were missed in the OVX group.

The results of quantitative micro-CT assessment indicate no statistical difference (*p* > 0.05) in bone microarchitectural parameters between the OVX and sham groups (Figures 4(c)–4(h)).

## 4. Discussion

This study focused on the knee joint, especially the tibia, and applied micro-CT and quantitative ultrasound to evaluate alterations in the bone and articular cartilage in an OVX rat model. The primary finding of this study was that significant changes in acoustic parameters of articular cartilage were detected in the rats with ovariectomy, whereas there were insignificant changes in microarchitecture of the cancellous bone. These results indicated that although both articular cartilage and bone are target tissues of estrogens, the degradation of the articular cartilage induced by estrogen deficiency occurred earlier and its characterizations could be detected by quantitative ultrasound. This study provides evidence to previous study suggesting that cartilage degradation might occur in an early stage of low estrogen levels [24].

The cartilage results of this study are similar to those of previous study showing that disuse-induced significant changes in the cartilage tissue [25]. However, disuse simultaneously induced cartilage degradation and bone loss [26]. This may be due to the fact that the articular cartilage and bone are subjected to high loads under gravitation force during daily walking, running, and jumping. Loading is important for keeping knees healthy. Therefore, the two tissues are sensitive to disuse or unloading. Although previous studies reported that estrogens affect the synthesis of both articular cartilage and cancellous bone through the receptor activator nuclear factor-kappa B ligand (RANKL) signaling pathway [27, 28], the present study suggested a rapid response of the articular cartilage to estrogen deficiency.

The definite relationship between OP and OA still expected more efforts. Herrero-Beaumont et al. reported that both high and low BMD conditions might initiate cartilage degradation in OA [14]. In this study, no significant alterations in the cancellous bone in the 3 wk OVX rats were found, whereas cartilage degradation was shown. Furthermore, a tendency of deterioration in the trabecular bone was shown in Figure 4, which is consistent with the findings (a decrease in BV/TV and Tb.N with an increase in Tb.Sp and SMI) of previous studies discovered in the 4 wk OVX rats [29], 6 wk OVX rats [30], and 12 wk OVX rats [19]. Brouwers et al. [31], however, showed significant deterioration in BV/TV, Tb.N, and Tb.Th in the 1 wk OVX rats. One possible cause of this divergence might be different ages, weight, and species of the investigated animals. 10-month-old Sprague-Dawley rats used in this study were in an old stage similar to the 50–60-year-old women, whereas 5.5-month rats were young adult rats. The findings of previous studies and this study implicate that the sudden estrogen deficiency after ovariectomy may have dissimilar significant effect on bone structure in the elderly rats to that in adult rats.

The present study has some limitations. Firstly, this study was limited in the detection of OP and OA occurred at the proximal tibia. Because OP and OA may occur at different joints and mechanisms of OP and OA are complex, the findings of previous studies are debatable, indicating that a defined relationship between OP and OA has not yet clearly delineated so far. Therefore, this study preliminarily focused on the tibia in the knee joint finding that the early effect of estrogen deficiency on the articular cartilage was faster detected using quantitative ultrasound than that on the cancellous bone detected using micro-CT. Secondly, in consideration of the relatively small sample size of each experimental group, nonparametric analyses were used in this study to reduce some impacts on the statistical results. In future, further studies with more samples are expected on the basis of the preliminary result of this study. The other limitation of this study is one time point. In the light of previous studies showing that the bone loss occurred in a relative late-stage (4 weeks after the ovariectomy operation) [19, 29, 30], this study aimed to detect the early alterations in tibial cartilage and cancellous bone 3 weeks after ovariectomy operation. However, according to our results, changes in the cartilage tissue at more time points such as 1 week or 2 weeks after ovariectomy are needed to be determined in future by quantitative ultrasound during the progression of menopause.

In conclusion, this study demonstrated that degradation of articular cartilage was detected earlier with quantitative ultrasound than that of the cancellous bone in 3 wk OVX rats. These results have implications that deterioration of the cartilage tissue after estrogen deficiency developed faster than cancellous bone. However, due to inconsistent findings of OP and OA, further studies are expected.
